# Meeting patient expectations: patient expectations and recovery after hip or knee surgery

**DOI:** 10.1007/s12306-017-0523-7

**Published:** 2017-11-22

**Authors:** B. Wiering, D. de Boer, D. Delnoij

**Affiliations:** 1 Tranzo (Scientific Centre for Transformation in Care and Welfare), Tilburg University, PO Box 90153, 5000 LE Tilburg, The Netherlands; 2 NIVEL (Netherlands Institute for Health Services Research), Utrecht, The Netherlands

**Keywords:** Patient-reported outcome measures, Hip and knee surgery, Patient preferences, Patient expectations

## Abstract

**Background:**

Although patient-centred care could help increase the value of healthcare, practice variations in hip and knee surgery suggest that physicians guide clinical decisions more than patients do. This raises the question whether treatment outcomes still meet patients’ expectations. This study investigated whether treatment outcomes measured by patient-reported outcome measures fulfil patients’ main expectations (i.e. decreased pain or improved functioning).

**Methods:**

Patients who underwent hip or knee surgery in 20 Dutch hospitals in 2014 were invited to a survey consisting of the KOOS Physical Function Short Form or the HOOS Physical Function Short Form, the NRS pain and the EQ-5D. Patients were asked their main reason for surgery and whether the expectations regarding this reason were fulfilled.

**Results:**

A total of 2776 patients completed the survey. The most common reason for surgery was improved functioning (43.7%). Patients who were unable to choose between pain relief and improved functioning and patients who aimed for pain relief experienced more problems before surgery. However, patients who were unable to choose improved more than patients who wanted to improve their functioning on the NRS pain during use and the EQ-5D. More patients who aimed for pain relief felt that their expectations were fulfilled compared to other patients.

**Conclusions:**

Although an expectation for an outcome was not related to a greater improvement on that outcome, patient expectations were an indication of patients’ improvement due to surgery. Differences in expectation fulfilment may be due to unrealistic expectations. To achieve optimal value, tailoring treatment using patient preferences and managing patient expectations is vital.

## Background

Many countries reformed their healthcare system in order to contain costs and improve the quality and efficiency of the delivered health care [1, 2]. An important way to contain costs and improve the efficacy and quality of care is by increasing the value (i.e. outcomes achieved per monetary unit) of health care. Improving the value of the health care system would benefit patients, healthcare professionals, and healthcare organisations, while the healthcare system becomes more sustainable [3]. Patient-centred care, defined by the Institute of Medicine as care that is “respectful of and responsive to individual patient preferences, needs, and values, and ensuring that patient values guide all clinical decisions” [4], could play an important part in increasing the value of health care, as patient-centred care may decrease the number of procedures and cost and improve patient safety and outcomes [5]. Patient-centred care is especially important for preference sensitive conditions, as there are usually several suitable treatments available, and the choice should depend on how patients value the pros and cons associated with the treatment options [6].

A good example of a common preference sensitive treatment is joint replacement, as there are several suitable alternatives for joint replacement, such as pain medication, exercise, and physical aids [7]. However, it is unclear whether patient-centred care is practiced when joint replacement is considered. An indication of a lack of patient-centred care can be found in the number of joint replacements, which are, especially for knee and hip replacements, known to vary hugely within and between countries [8–10]. Practice variations are often partly seen as a variation in physician’s preferences [11, 12], while for patient-centred care, it is very important whether the treatment corresponds with the patient’s preferences [5]. Research indicates that better informing and guiding patients by shared decision making (i.e. more patient-centred care) reduces variation between hospitals [13].

Another indication of a lack of patient-centred care is that research suggests that many patients would not opt for joint replacement if they were informed of the evidence base [14], or if their decision-making process was supported by a decision aid [15]. Respecting patients’ preferences and allowing patients to make an informed choice is essential to patient-centred care. As many informed patients would not choose joint replacement as their preferred treatment [14, 15] and more patient-centred care should reduce variation between hospitals [13], the great number of joint replacements taking place every year [16] and the variations in the number of joint replacements can not only be explained by the varying preferences of well-informed patients. In view of increasing the value of health care, this possible lack of patient-centred care raises the question whether optimal quality of care is still achieved for hip and knee replacements. Even if patient-centred care was not practiced, does treatment still result in good outcomes which match patients’ preferences and expectations for certain outcomes?

To give more insight into whether undergoing joint replacement surgery meets patients’ preferences and expectations for a certain outcome, a specific case was used. Patients who underwent hip or knee surgery were asked to complete patient-reported outcome measures (PROMs), importance ratings and a closed question regarding the main reason for surgery. As earlier research showed that patients have especially high expectations for pain relief and function improvement, we limited the answer options to either pain relief or function improvement [17, 18]. By matching PROMs for the various outcomes, importance ratings per outcome and the patients’ primary reasons for surgery, the present study aimed to investigate whether the treatment results match the patients’ expectations by answering the following research questions:

1. What do patients expect to achieve by undergoing hip or knee surgery?
2. Do patients who aim for improved functioning or pain relief actually show improved functioning or a decrease in pain after surgery?
3. Do patients think that their expectations regarding their main reason for surgery were met?

## Materials and methods

### Participants

This study is part of a bigger study carried out by a collaboration of Dutch health insurers [19]. Patients who underwent hip or knee surgery in 20 hospitals in the Netherlands were invited within 12 months of their treatment to fill in a questionnaire. Recruitment took place between December 2014 and February 2015. Patients younger than 16 years of age and patients who were already invited for a similar questionnaire earlier that year were excluded. Patients were invited to complete one survey regardless of the number of times they underwent hip and/or knee surgery. No selection based on the medical reason for surgery was made (e.g. a fractured hip, knee injury, arthritis). Hospital selection was based on the average and more or less even number of patients undergoing hip and knee replacement. Hospitals which invited patients themselves were excluded.

### Procedure

As part of their objective to contract healthcare professionals based on quality of care and price [20], health insurers in the Netherlands may within certain boundaries legally ask clients to participate in research with the aim of improving the quality of care. This type of research does not fall under the Dutch Medical Research Involving Human Subjects Act (WMO), and a formal ethical board review is therefore not required [21]. The data were collected according to the guidelines provided by the National Health Care Institute. The guidelines concern both informed consent and privacy [22]. Participation is anonymous and voluntary. To ensure anonymity names are converted to unique survey numbers. This also prevents the inclusion of double surveys in the data set. Furthermore, hospitals do not have access to the data on an individual patient level. Additionally, health insurance in the Netherlands is open to everyone and health insurers are not allowed to select their clients, or adjust premiums and/or cover based on individual clients. Patients’ answers therefore have no impact on the care they receive from the hospital, the insurance premiums they pay or the insurance cover they receive.

For some treatments clients receive a letter within 12 months after surgery inviting them to complete a questionnaire regarding their perspective on their hospital stay and treatment. For the present study, rating scales and several additional questions regarding expectations were added to this questionnaire. The Dillman method was used for contacting clients [23]. Clients were asked to send a card back, which was enclosed with the letter, if they did not wish to participate. A week after the first letter a reminder was sent. Two weeks after the first reminder, a reminder and a paper version of the questionnaire was sent. Three weeks later a final reminder was sent.

### Measures

For this study parts of the questionnaire containing basic information, four PROMs and questions regarding the patients’ main reason for surgery were used. The basic information included in this study concerned gender, age, overall health, whether complications were experienced, the duration of experienced function limitations before surgery (shorter or longer than six months), overall psychological health, and education level.

The PROMs consisted of the HOOS Physical Function Short Form (HOOS-PS) [24], KOOS Physical Function Short Form (KOOS-PS) [25], the EQ-5D [26] and the NRS pain [27]. Patients received the HOOS-PS or the KOOS-PS depending on whether the patient underwent hip or knee surgery. The HOOS-PS and the KOOS-PS are short measures of physical functioning level, where the degree of difficulty that was experienced due to hip or knee problems can be rated on a five-point scale (‘None’- ‘Extreme’). Both the HOOS-PS and the KOOS-PS are validated PROMs [28–31]. The HOOS-PS consists of five items. The KOOS-PS consists of seven items. For the total score the individual item scores were added up. The corresponding Rasch-based person interval level score can be found in the papers by Davis et al. [24] and Perruccio et al. [25]. A higher score indicated a lower functioning level. Both PROMs were asked twice using a then-test, where respondents answer the questionnaires for how they perceived themselves to have been last month and a month prior to surgery [32].

The second PROM concerned a validated health status questionnaire, the EQ-5D [26]. This questionnaire comprises three statements (‘No problems’- ‘Major problems’) about five dimensions of health status. Patients indicated which statement fitted their situation best, both at the time of measurement and just before surgery. The EQ-5D index scores were calculated based on general population valuation surveys [33]. A higher score indicated a better health status.

The last PROM was the NRS pain. The NRS pain is a validated numerical rating scale where patients can rate the intensity of their pain from 0 to 10 (‘No pain’- ‘Worst possible pain’) [27]. Patients rated their pain intensity during rest and while using their hip or knee for both the month prior to surgery and the last month.

Patients were asked what their main reason for undergoing surgery was. Answer options were: ‘Mostly to reduce the pain’, ‘Mostly to improve function’, and ‘I cannot choose’. The answer options are supported by earlier research [34]. Additionally, our earlier study where patients rated the importance of several PROMs showed that patients who chose functioning as their main reason for undergoing surgery, considered many items from the HOOS-PS and the KOOS-PS more important than patients who chose pain relief [35]. Patients who chose pain relief considered the item pain/discomfort from the EQ-5D more important than patients who chose function improvement. The main reasons for undergoing surgery appear to be reflected in patients’ preferences for PROM items. Additionally, we asked patients using an open question what their main reason for undergoing surgery was. As the answers to this question mainly concerned pain and function, we used the closed question.

Patients were also asked whether they thought that their expectations regarding their main reason for surgery were met. Answer options were: ‘Yes’, ‘Partly’ and ‘No’.

### Statistical analyses

Univariate analyses were used to describe the sample regarding socio-demographics and PROM scores. To give insight into whether patients who preferred to improve their functioning or decrease their pain level improved on their preferred outcome after surgery, a series of linear regressions were used.^1^ The dependent variable was the mean difference between the pre and post operation score of either the HOOS-PS, KOOS-PS, NRS or EQ-5D. The independent variable was the patients’ main reason for undergoing surgery. As univariate analyses indicated group differences in the PROM pre-scores, analyses were controlled for the pre-score of the PROM used as a dependent variable. Analyses were also controlled for the casemix variables education, age, sex, overall health, complications, the duration of experienced function limitations before surgery and the number of days between surgery and questionnaire completion, as the level of pain and functioning changes over time [36]. Similar analyses were performed to investigate the influence of the main reason for surgery on pre- and post-PROM scores. No scale ceiling effects were found. Finally, a Chi-square test was conducted to analyse whether patients who chose a certain reason for surgery differed in whether they felt that their expectations were fulfilled. Analyses were performed using SPSS 22 [37].

## Results

### Response

A total of 3996 patients received an invitation to the survey. The questionnaire was completed online by 1108 patients, while 1811 patients used the paper version. A total of 1077 patients did not complete the questionnaire, of which 488 patients declined to participate by sending back the card. Forty patients were excluded as their questionnaire was completed by someone else. One hundred and three patients were excluded because they answered less than five questions. This resulted in 2776 completed questionnaires. The questionnaire was completed on average 274.4 (SD = 70.2) days after surgery. The only difference between non-respondents and respondents was an age difference (73.2 compared to 72.0 years; (*F*(1,3994) = 11.77, *p* = .00).

### Sample characteristics

Slightly more patients received hip surgery (52.5%) compared to knee surgery. The majority of patients were women (65,7%) and received secondary education (56.0%).The patients’ age was 72.0 years on average (Range = 28–98; SD = 9.1). Patients weighed on average 81.0 kilograms (Range: 41–183; SD: 15.4) and were 169.9 centimetres tall (range 140–198; SD: 9.0). Most patients (76.8%) noted an impact of their hip or knee problem on their daily life for longer than 6 months before surgery. 27 per cent of patients experienced complications after surgery. On average patients improved on all PROMs (Table 1). The most common reason for surgery was improved functioning (43.7%). Other answers were pain relief (34.8%) and I can not choose (9.8%).

**Table 1:** PROM scores and expectation fulfilment for patients who underwent surgery to improve their functioning, decrease their pain level or could not choose between either reasons

	Improved functioning		Decreased pain level		I can not choose	
	*N*	Mean (SD)	*N*	Mean (SD)	*N*	Mean (SD)
HOOS-PS score before surgery	449	53 (21.1)	413	59.8 (22.8)	113	60.8 (21.9)
HOOS-PS score after surgery	454	24.5 (18.8)	421	25.6 (21.3)	113	22 (19.8)
HOOS-PS change score	424	28.9 (25.8)	390	35.4 (25.9)	109	40.2 (27.5)
KOOS-PS score before surgery	524	52.9 (20.1)	369	53.3 (21.3)	112	62 (24.5)
KOOS-PS score after surgery	548	33 (18.3)	378	35.8 (17.4)	118	35.5 (20.1)
KOOS-PS change score	507	20.3 (24.2)	356	17.6 (24.9)	110	27.4 (27.6)
EQ-5D score before surgery^a^	945	.51(.31)	752	.37 (.31)	226	.34 (.31)
EQ-5D score after surgery^a^	945	.84 (.21)	765	.83 (.22)	229	.84 (.20)
EQ-5D change score	873	.34 (.34)	703	.46 (.34)	216	.52 (.32)
NRS pain during rest before surgery	1109	6.5 (2.4)	902	7.4 (2.4)	250	7.5 (2.5)
NRS pain during rest after surgery	1140	2.4 (2.8)	912	2.5 (3)	260	2.3 (2.8)
NRS Pain during rest change score	1086	4.2 (3.6)	878	5 (3.7)	249	5.5 (3.2)
NRS pain during use before surgery	1117	7.4 (2.4)	900	8.2 (2)	249	8.4 (2.1)
NRS pain during use after surgery	1140	3 (2.9)	905	3.1 (3)	253	2.7 (2.7)
NRS pain during use change score	1093	4.6 (3.6)	875	5.3 (3.7)	244	6 (3.3)

		Per cent (%)		Per cent (%)		Per cent (%)
Expectations were met	765	63.1	694	71.8	160	58.8
Expectations were partly met	344	28.4	211	21.8	79	29.0
Expectations were not met	94	7.8	43	4.5	26	5.6

^a^For every PROM except the EQ-5D a lower score indicates better health. For the EQ-5D a higher score indicates better health

The relationship between the patients’ reasons for surgery and surgery outcomes.

**Fig. 1:**
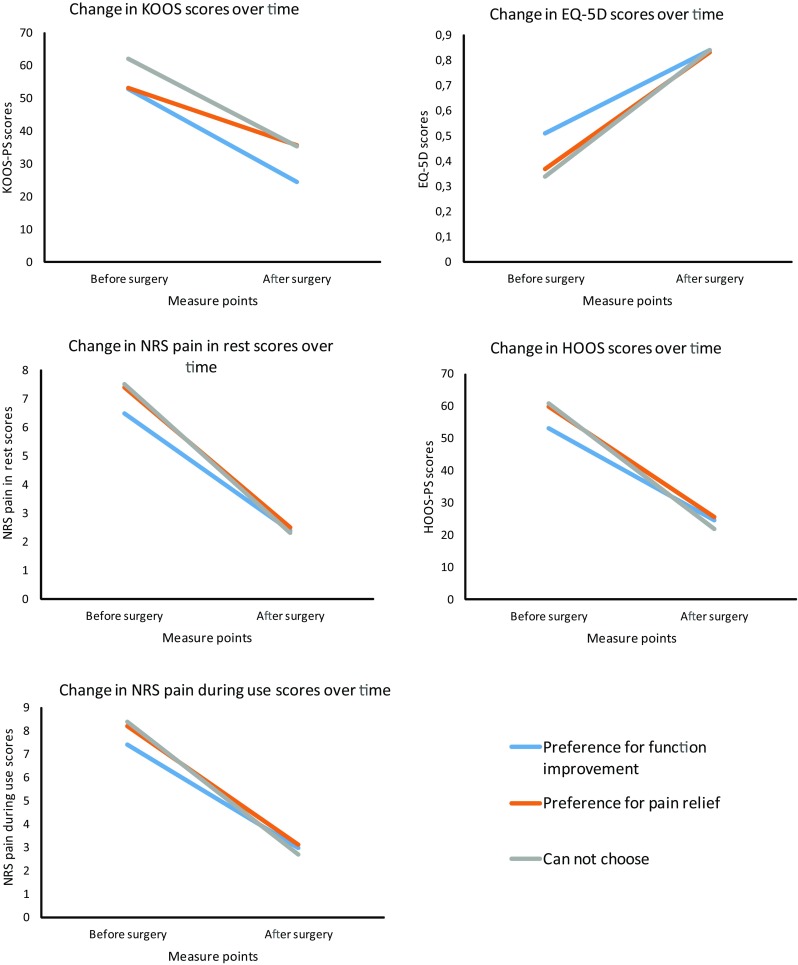
Improvement in PROM scores for patients who underwent surgery to improve their functioning, decrease their pain level or could not choose between either reasons after surgery. For all PROMs except the EQ-5D a lower score means less pain or better functioning

Patients who could not choose between reasons for surgery and patients who aimed for pain relief scored less well on PROMs before surgery (Table 2). Improvement on PROM scores was mainly dependent upon whether patients experienced complications, age, education level, time between surgery and questionnaire completion, the duration of experienced limitations before surgery and overall health (Table 3). Patients who underwent surgery mainly to improve their functioning level did not improve significantly better than patients who mainly chose to have surgery for pain relief. However, if patients were unable to choose between decreased pain and improved functioning, they improved more than patients who wanted to improve their functioning on the NRS pain during use and the EQ-5D (Table 3). All patients achieved the same after-surgery PROM scores, except for the NRS pain during use and the EQ-5D. Patients who could not choose achieved a better health status and experienced less pain while using their hip or knee after surgery than patients who wanted to improve their functioning (Table 4, Fig. 1).

**Table 2:** Factors related to the before surgery scores on the HOOS-PS (hip functioning level), KOOS-PS (knee functioning level), NRS pain during use, NRS pain during rest and the EQ-5D (health status)

	HOOS-PS (*N* = 920)		KOOS-PS (*N* = 948)		NRS pain during use (*N* = 2129)		NRS pain during rest (*N* = 2125)		EQ-5D (*N* = 1831)	
	*β*	*P*	*β*	*P*	*β*	*P*	*β*	*P*	*β*	*P*
*Reason for surgery: function (ref)*										
*Pain*	.13	.00	− .02	.49	.15	.00	.15	.00	− .18	.00
Unable to choose between reasons	.10	.00	.11	.00	.11	.00	.10	.00	-.15	.00
Complications	− .05	.15	.01	.85	.00	.84	.01	.60	.01	.71
Age	− .12	.00	− .12	.00	− .16	.00	− .14	.00	.16	.00
Sex	.15	.00	.09	.01	.10	.00	.07	.00	− .09	.00
*Education: lower to middle vocational education (ref)*										
> High school level	− .00	.99	− .00	.94	− .02	.54	.06	.03	− .05	.09
High school/secondary education	− .09	.06	.00	.93	− .02	.43	.02	.42	.02	.60
Higher vocational education (BSc)	− .12	.00	− .07	.08	− .05	.04	− .10	.00	.06	.02
University (BSc/MSc)	− .07	.05	− .06	.07	− .04	.08	− .05	.02	.05	.03
Overall health	.11	.00	.13	.00	.06	.01	.07	.00	− .15	.00
Type of surgery (hip or knee)^a^	–	–	–	–	− .01	.52	− .04	.10	.05	.05
Number of days after surgery	.07	.02	− .05	.14	− .02	.31	.01	.53	.06	.01
Duration of function limitations before surgery	.14	.00	.08	.01	.14	.00	.11	.00	− .05	.02

^a^Type of surgery was only included for PROMs which were completed by both hip and knee surgery patients

**Table 3:** Factors related to the change scores from the HOOS-PS (hip functioning level), KOOS-PS (knee functioning level), NRS pain during use, NRS pain during rest and the EQ-5D (health status)

	HOOS-PS change score (*N* = 877)		KOOS-PS change score (*N* = 920)		NRS pain during use change score (*N* = 2086)		NRS pain during rest change score (*N* = 2091)		EQ-5D change score (*N* = 1713)	
	*β*	*P*	*β*	*P*	*β*	*P*	*β*	*P*	*β*	*P*
HOOS-PS pre-score^a^	.75	.00								
KOOS-PS pre-score^a^			.77	.00						
NRS pain during use pre-score^a^					.65	.00				
NRS pain during rest pre-score^a^							.68	.00		
EQ-5D pre-score^a^									− .85	.00
*Reason for surgery: function (ref)*										
Pain	.02	.35	− .03	.15	− .00	.93	− .01	.73	.01	.32
Unable to choose between reasons	.04	.13	.01	.70	.04	.04	.03	.07	.04	.01
Complications	.03	.19	.07	.00	.06	.00	.06	.00	.06	.00
Age	− .11	.00	− .07	.00	− .03	.09	− .05	.00	− .05	.00
Sex	− .02	.44	− .05	.01	− .02	.37	.01	.38	− .02	.07
*Education: Lower to middle vocational education (ref)*										
> High school level	− .08	.01	.00	.87	− .02	.31	− .04	.03	− .04	.02
High school/secondary education	.01	.89	− .01	.78	.01	.83	− .02	.28	− .01	.44
Higher vocational education (BSc)	.07	.02	− .02	.48	.07	.00	.06	.00	.01	.51
University (BSc/MSc)	.04	.10	.00	.88	.05	.01	.05	.01	.01	.47
Overall health	− .20	.00	− .20	.00	− .16	.00	− .12	.00	− .18	.00
Number of days after surgery	.00	.95	.08	.00	.06	.00	.06	.00	.01	.29
Type of surgery (hip or knee)^b^	–	–	–	–	− .08	.00	− .08	.00	− .02	.13
Duration of function limitations before surgery	− .05	.04	− .06	.01	− .06	.00	− .05	.01	− .05	.00

^a^Analyses were controlled for the PROM pre-score. Although all pre-scores are included in the table, only the pre-score corresponding with the dependent variable was included

^b^Type of surgery was only included for PROMs which were completed by both hip and knee surgery patients

**Table 4:** Factors related to the after surgery scores on the HOOS-PS (hip functioning level), KOOS-PS (knee functioning level), NRS pain during use, NRS pain during rest and the EQ-5D (health status)

	HOOS-PS (*N* = 877)		KOOS-PS (*N* = 920)		NRS pain during use (*N* = 2086)		NRS pain during rest (*N* = 2091)		EQ-5D(*N* = 1713)	
	*β*	*P*	*β*	*P*	*β*	*P*	*β*	*P*	*β*	*P*
HOOS-PS pre-score^a^	.13	.00								
KOOS-PS pre-score^a^			.12	.00						
NRS pain during use pre-score^a^					− .04	.09				
NRS pain during rest pre-score^a^							.07	.00		
EQ-5D pre-score^a^									.10	.00
*Reason for surgery: function (ref)*										
Pain	− .03	.35	.05	.15	.00	.93	.01	.73	.02	.32
Unable to choose between reasons	− .05	.13	− .01	.70	− .05	.04	− .04	.07	.07	.01
Complications	− .04	.19	− .10	.00	− .07	.00	− .08	.00	.10	.00
Age	.15	.00	.10	.00	.04	.09	.07	.00	− .08	.00
Sex	.03	.44	.08	.01	.02	.37	− .02	.38	− .04	.07
*Education: lower to middle vocational education (ref)*										
> High school level	.11	.01	− .01	.87	.03	.31	.06	.03	− .06	.02
High school/secondary education	− .01	.89	.01	.78	− .01	.83	.03	.28	− .02	.44
Higher vocational education (BSc)	− .09	.02	.03	.48	− .09	.00	− .08	.00	.02	.51
University (BSc/MSc)	− .05	.10	− .01	.88	− .06	.01	− .06	.01	.02	.47
Overall health	.28	.00	.29	.00	.21	.00	.16	.00	− .31	.00
Number of days after surgery	− .00	.95	− .11	.00	− .08	.00	− .08	.00	.02	.29
Type of surgery (hip or knee)^b^	–	–	–	–	.10	.00	.10	.00	− .03	.13
Duration of function limitations before surgery	.07	.04	.08	.01	.08	.00	.06	.01	− .08	.00

^a^Analyses were controlled for the PROM pre-score. Although all pre-scores are included in the table, only the pre-score corresponding with the dependent variable was included

^b^Type of surgery was only included for PROMs which were completed by both hip and knee surgery patients

### Expectation fulfilment

Patients who chose one reason for undergoing surgery significantly differed from patients who chose a different reason in whether they felt that their expectations were fulfilled (*χ* ^2^ (4) = 31.42, *p* = .00). Patients who wished for pain relief answered significantly more often that their expectations were met than patients who aimed for function improvement (*χ* ^2^ (2) = 24.43, *p* = .00) or patients who were unable to choose between pain and function (*χ* ^2^ (2) = 19.92, *p* = .00).

## Discussion

The present study aimed to investigate whether the outcomes of joint replacement surgery meet patients’ preferences and expectations for certain outcomes. This study therefore first investigated what the patients’ main reason (i.e. decreased pain or improved function) for surgery was. Although patients were given the option to indicate that they were not able to choose between pain relief and function improvement, most patients were able to give one clear reason. Both decreasing pain and improving functioning were common reasons for surgery. However, the majority of both hip and knee surgery patients were primarily focused on improving their functioning. Earlier research among patients undergoing knee surgery indicated that pain relief is the most common reason for joint replacement [34]. However, the patients in this earlier study were asked their reason for surgery using an open question. Although pain is something patients are confronted with every day, function may be a slightly more abstract term. Some other common answers were the inability to carry out usual activities, social and recreational activities or work. All these limitations can occur because of both pain and function loss. Furthermore, other research indicated that patients have similar high expectations for both physical activities such as improved walking ability and pain relief [17, 18], suggesting that aspects of function improvement were also important to patients in other studies.

As most patients clearly chose for either improved functioning or pain relief, we also investigated whether patient preferences are an indication of surgery results. It appears that patient preferences can be an indication of the patients’ physical problems, pain and health status before surgery and the possibility to improve on these outcomes after surgery. Analyses show that a preference for a certain outcome was not related to a greater improvement on this outcome. However, this study did find significant differences between the patients who chose different reasons for surgery in both starting point and improvement after surgery. Both patients who could not choose between pain relief and function improvement and patients who aimed for pain relief started out worse than patients who chose function improvement as their main reason for surgery. Remarkably, the patients who were unable to choose improved more than the other two patient groups on pain and health status. This improvement ensured that the after-surgery PROM scores for these patients were similar or even better than the PROM scores of the other patient groups. Earlier research showed that being less healthy is strongly related to less improvement after surgery [38]. The present study showed that this does not always has to be the case, as patient expectations can be an indication of how much patients will benefit from surgery regardless of their health before surgery.

Finally, as patient expectations appear to be related to surgery success, this study investigated whether patients with certain expectations regarding their main reason for surgery felt that these expectations were met. Remarkably, although surgery resulted in similar results for patients who aimed for pain relief compared to the other patient groups, significantly more patients felt that their expectations were met. On the other hand, although patients who were unable to choose between reasons benefitted far more from surgery, an equal number of patients felt that their expectations were met compared with patients who wished for function improvement. Further research is needed to investigate why some patients feel that their expectations are met and others do not, regardless of surgery results.

### Limitations

Some study limitations should be taken into account. First, a retrospective post-then-pre design was used to conduct this study. This may have biased the patients’ recall of their health before surgery. However, differences between measuring before and after surgery and only afterwards appear to be minimal [39]. Results may even be more accurate because of the lack of response shift [40]. Another factor which may result in recall bias is the fact that although participants underwent surgery sometime during the year 2014, patients were invited to participate at one time point. The time between surgery and survey completion varies therefore greatly between patients. To ensure that these differences in time had as little impact as possible, analyses were controlled for the number of days between surgery and questionnaire completion. Furthermore, patients’ experiences and answers could also have been influenced by having to pay a deductible excess towards the surgery. Patients in the Netherlands pay a compulsory monthly insurance premium which covers most health care and is unrelated to how much health care the patient uses. Besides the monthly insurance premium, patients pay a compulsory deductible excess if they use health care. This excess is the same standard amount for everyone and is deductible on almost all health care claims. Patients with a lower income will receive subsidies to help fund their health care [41]. Additionally, although we investigated several differences between patient groups who chose a certain reason for surgery, we were limited by the data we gathered. There may be other reasons why the ‘I can not choose’ group started out worse and improved much more than the other groups. Finally, although the scales did not have a ceiling effect, patients may have had their own ‘ceiling effect’, as their health or circumstances may have limited how much they could improve.

### Implications

The present results indicate that most patients improve after hip or knee surgery. As patients who undergo surgery for certain reasons differ in both pre-surgery health and the level of improvement after surgery, the level of surgery success seems to be associated with patients’ expectations regarding a certain outcome. If patient expectations for a certain outcome to a certain extent ‘predict’ the patient-reported success of the surgery, taking into account patient preferences and expectations during the decision-making process for treatment may be important for several reasons. First, patients would benefit if their preferences and expectations are taken into account when making a decision regarding treatment as they would receive a treatment better tailored to their personal needs. Second, as preferring a certain outcome is not directly related to greater improvement on this outcome, and there is some research that shows that patients can entertain unrealistic expectations [42], discussing patient expectations may help manage patients’ expectations. This would not only help guard for disappointment but would perhaps help patients make a more suitable choice regarding treatment. Third, if patient expectations are met and patients receive fitting treatment, this may also help improve the value of our health care [5]. Research, however, indicates that in practice patient expectations are rarely discussed [43]. Training physicians may help solve this problem, as research indicates that physicians who were adequately trained elicited patient expectations more often [43].

Training professionals to practice more patient-centred care may not only improve patient outcomes, but may also improve expectation fulfilment. Our results show that not all patients felt that their expectations regarding surgery were met and that expectation fulfilment appears to be unrelated to improvement. Although better expectation management may help decrease the number of unsatisfied patients, less expectation fulfilment may also be due to a lack of patient-centred care. A more patient-centred approach may help patients receive a treatment which suits their preferences and needs. This may increase the number of patients who feel that their expectations are fulfilled by the treatment they received.

Finally, patients who were unable to choose between reasons for surgery improved more than other patients on both the NRS pain during use and the EQ-5D and maintained this lead after surgery. It would be interesting to investigate what makes surgery such a success for these patients. The results of such an investigation may be helpful in achieving better surgery results for every patient.

## Conclusion

Patients’ main reason for surgery was improved functioning. Although expectations regarding a certain outcome were not related to greater improvement on that outcome, surgery success may still be associated with patient expectations, as patient expectations were an indication of patients' improvement due to surgery. Patients who could not choose between reasons for surgery improved significantly more on pain during use and health status measures, compared to patients who aimed for improved functioning, while no difference was found between patients who expected pain reduction and patients who expected improved function. However, these surgery results did not translate into fulfilled expectations. Remarkably, more patients who aimed for pain relief felt that their expectations were met than patients who chose to have surgery to improve their functioning or to improve both pain relief and function. To achieve optimal value, patient preferences and expectations need to be taken into account while deciding for a treatment. Additionally, patient expectations need to be managed to ensure that patients choose the right treatment and know what to expect regarding surgery results.

## Footnotes

1 A series of multilevel models confirmed that the hospital variance of PROM change scores was virtually negligible in the context of the current analyses; Hospital level variance was significant for two PROM items only. Accordingly, single-level regression models are reported in the present paper for ease of presentation.
